# Silk genes and silk gene expression in the spider *Tengella perfuga* (Zoropsidae), including a potential cribellar spidroin (CrSp)

**DOI:** 10.1371/journal.pone.0203563

**Published:** 2018-09-20

**Authors:** Sandra M. Correa-Garhwal, R. Crystal Chaw, Thomas H. Clarke, Liliana G. Alaniz, Fanny S. Chan, Rachael E. Alfaro, Cheryl Y. Hayashi

**Affiliations:** 1 Department of Evolution, Ecology, and Organismal Biology, University of California, Riverside, California, United States of America; 2 Department of Neurology, Oregon Health and Science University, Portland, Oregon, United States of America; 3 J. Craig Venter Institute, Rockville, Maryland, United States of America; 4 Division of Arthropods, Museum of Southwestern Biology, Albuquerque, New Mexico, United States of America; 5 Division of Invertebrate Zoology and Sackler Institute for Comparative Genomics, American Museum of Natural History, New York, New York, United States of America; Charles University, CZECH REPUBLIC

## Abstract

Most spiders spin multiple types of silk, including silks for reproduction, prey capture, and draglines. Spiders are a megadiverse group and the majority of spider silks remain uncharacterized. For example, nothing is known about the silk molecules of *Tengella perfuga*, a spider that spins sheet webs lined with cribellar silk. Cribellar silk is a type of adhesive capture thread composed of numerous fibrils that originate from a specialized plate-like spinning organ called the cribellum. The predominant components of spider silks are spidroins, members of a protein family synthesized in silk glands. Here, we use silk gland RNA-Seq and cDNA libraries to infer *T*. *perfuga* silks at the protein level. We show that *T*. *perfuga* spiders express 13 silk transcripts representing at least five categories of spider silk proteins (spidroins). One category is a candidate for cribellar silk and is thus named cribellar spidroin (CrSp). Studies of ontogenetic changes in web construction and spigot morphology in *T*. *perfuga* have documented that after sexual maturation, *T*. *perfuga* females continue to make capture webs but males halt web maintenance and cease spinning cribellar silk. Consistent with these observations, our candidate CrSp was expressed only in females. The other four spidroin categories correspond to paralogs of aciniform, ampullate, pyriform, and tubuliform spidroins. These spidroins are associated with egg sac and web construction. Except for the tubuliform spidroin, the spidroins from *T*. *perfuga* contain novel combinations of amino acid sequence motifs that have not been observed before in these spidroin types. Characterization of *T*. *perfuga* silk genes, particularly CrSp, expand the diversity of the spidroin family and inspire new structure/function hypotheses.

## Introduction

Spiders are widely distributed and abundant in most terrestrial communities, and their evolutionary success is partly associated with diversification of silk usage [1–3]. Silk is an important feature of spider biology, and all spiders produce silk for an array of essential, fitness-related tasks including prey capture, reproduction, locomotion, and protection of progeny [4]. Most of the studies on spider silk use and molecular composition have been heavily focused on cob-web and ecribellate orb-web weaving spiders. These spiders have several silk types, including aciniform, major ampullate, pyriform, and tubuliform silks. However, there are many other types of spiders with different combinations of silk types. For example, *Tengella perfuga*, Dahl 1901 (Zoropsidae) uses copious amounts of silk to build sheet webs with deep retreats in high elevation remnant cloud forest habitats in Nicaragua [5,6]. *T*. *perfuga* spiders belong to the RTA (retrolateral tibial apophysis) clade, which diverged approximately 191–247 million years ago from orb-web weaving spiders, and are cribellate spiders [7,8]. Cribellate spiders have one pair of silk spinnerets modified into a cribellum, a plate-like spinning organ that is dotted with numerous miniscule spigots. From this dense field of spigots, thousands of ultrafine fibrils are produced; this silk type is referred to as cribellar silk [9–12]. Cribellar silk has adhesive properties and is an important functional element of the prey-capture webs spun by cribellate spiders [13–15]. To achieve stickiness, cribellar silk uses a combination of van der Waals and hygroscopic forces as well as the absorption of epicuticular waxes of prey insects [13,14,16]. Cribellar silk is stiff yet extensible, while the core axial fiber can be stiffer than major ampullate fibers [17,18]. Cribellar fibrils work together to stretch up to 500% their original length [17].

Silk production in spiders involves a combination of highly specialized genes, structures, and behaviors. Spider silk genes are expressed in silk glands, which are located in the abdomen. Spider silk genes encode proteins known as spidroins (spidroin is a contraction of spider fibroin [19]), and the silk genes are members of the spidroin gene family [19–23]. Each silk gland has a distinct spidroin expression pattern, and a duct that leads to its own spigot located on the spinnerets [24,25]. Spider silk spigots are morphologically distinctive and are named according to the silk gland connected to them. From each spigot type, a functionally specific silk type emerges. For instance, pyriform spidroins are synthesized in pyriform glands, and pyriform silk fibers emerge from pyriform spigots [24,26].

*T*. *perfuga* spiders, with their large body size and ease of rearing in captivity, provide an opportunity to investigate the genetics of cribellar silk [5,6]. Additionally, ontogeny of silk usage and silk spigots in *T*. *perfuga* has recently been examined [6,27]. Adult female *T*. *perfuga* spiders use silk for foraging, building retreats, and constructing egg sacs. Spiderlings make small sheet webs without cribellar silk. As the spiderlings mature, their webs become more complex with the addition of cribellar silk.

Cribellar silk fibers fill the sheet of adult female webs, lining the retreat and knockdown lines that extend from the substrate to the sheet. By contrast, after becoming sexually mature, males abandon their webs and adopt a wandering life style. Based on scanning electron microscopy, spigots corresponding to aciniform, cribellate, major ampullate, minor ampullate, pyriform, and tubuliform silk glands have been imaged for *T*. *perfuga* [27]. Additionally, there are three spigots found on the posterior lateral spinnerets that are arranged together in a triad-like morphology that are connected to unidentified gland types. From these spigots, the largest spigot is called the “modified spigot,” and it is flanked by two smaller spigots. This spigot trio has been designated the MS-FL triad, or “modified spigot with flankers” triad by Alfaro et al [27,28]. The arrangement of the MS-FL spigots is quite similar to the spigot triad found in araneoids. The araneoid triad produces sticky capture lines, and is comprised of a flagelliform gland spigot that produces the axial line and two aggregate gland spigots that produce glue-like silk [29]. In *T*. *perfuga*, the “modified spigot” produces the axial lines the cribellar silk is combed out on [28]. Changes in *T*. *perfuga* spigot ontogeny also involve the cribellum. With successive molts, the number of cribellar spigots and size of the cribellum increases as the spiders molt to adulthood, except that males lose their cribellar spigots in the final molt [27].

Here, we use expression libraries to characterize the silk genes of *T*. *perfuga*. Based on studies of their silk usage and silk spigot ontogeny [6,26,27], we hypothesize that *T*. *perfuga* will express spidroin genes with orthology to known aciniform, major ampullate, minor ampullate, pyriform, and tubuliform silk genes. If true, then *T*. *perfuga* spidroins will group with corresponding orthologous genes in phylogenetic analyses. Additionally, because *T*. *perfuga* uses extensive amounts of cribellar silk for capture web construction, we hypothesize that there will be an additional spidroin that is a candidate constituent of cribellar silk. To our knowledge, a cribellar silk spidroin has yet to be described at the molecular level. Because mature males lose the ability to produce cribellar silk, we would expect mature females but not mature males to express this spidroin. Finally, we predict that the main silk genes associated with capture web construction will be highly expressed compared to other spidroin genes because *T*. *perfuga* spiders use copious amounts of silk in their capture webs.

## Materials and methods

### cDNA library construction and sequencing

All spiders used in this study were part of a lab-reared spider colony. The colony was started with mature spiders collected in Nicaragua (Selva Negra, 12.9984^o^N, 85.9105^o^W) in May 2012 and 2014 [6]. *T*. *perfuga* adult individuals were used for all silk gland dissections. *T*. *perfuga* adult individuals were used for all silk gland dissections. Spiders were anesthetized with CO_2_ and euthanized by separating the cephalothorax from the abdomen. Immediately after euthanization, silk glands were dissected from each individual, flash frozen in liquid nitrogen, and stored at -80°C. From the total silk gland complement of a *T*. *perfuga* spider, the following silk glands were identified and dissected based on shape, size, and position: ampullate-shaped, tubuliform-shaped (present in females only), and an assortment of small silk glands which were close to and left attached to the spinnerets. These small silk glands were presumed to include the cribellate silk glands.

Using the ampullate-shaped silk glands and all the smaller silk glands from mature male and female spiders; and the tubuliform silk glands from mature females, silk gland type-specific plasmid-based cDNA libraries were constructed and screened following the methods described in Garb et al. [30]. The libraries were screened with γ-^32^P-labeled oligonucleotide probes designed from previously characterized spidroins [31,32]. To discover novel spidroins, about one third of each library was screened for size, and clones with inserts > 600 base pairs were sequenced using T7 and SP6 universal primers. BLASTX searches revealed that the sequenced cDNAs included 30 spidroin clones. Each spidroin clone contained repetitive region and the conserved coding region for the C-terminal domain. One clone, a tubuliform spidroin (*T*. *per*_TuSp_C), was fully sequenced (2,971 base pairs) using the transposon-based EZ-Tn5 <TET-1> insertion kit (Epicentre). *T*. *perfuga* cDNA clones were Sanger sequenced at the University of California Riverside (UCR) Genomics Core Facility.

### RNA-Seq library construction, sequencing, and assembly

The total set of silk glands was dissected from each of two *T*. *perfuga* females raised by R. Alfaro. The glands were flash frozen in liquid nitrogen and stored at -80°C. Separate RNA extractions were done for the total set of silk glands from each individual spider following the methods of Starrett et al. [33]. In short, total RNA was extracted from each individual using TRIzol (Invitrogen) and purified with an RNeasy mini kit (Qiagen). Two RNA-Seq libraries were then made from cDNA prepared using the method described in Starrett et al. [33] with the modification that first strand cDNA was primed with both oligo-d(T) and random hexamers. Indexed libraries were constructed from the cDNA with the Encore NGS Library System (NuGen). Sequencing (paired end, 100 cycles each) was done on a HiSeq System (Illumina) at the UCR Genomics Core Facility.

Raw sequencing reads from each FASTQ file were processed by clipping the adaptors and removing low quality reads with Trimmomatic [34]. Quality of resulting filtered reads was assessed using FastQC (Babraham Bioinformatics FastQC Package). All *T*. *perfuga* reads were combined to assemble a *de novo* female silk gland transcriptome with Trinity v2.1.1 using default parameters [35]. See S1 Table for assembly statistics. Quality of the *T*. *perfuga* assembly was approximated using N50 and completeness determined by comparison to the arthropod v9 set of Universal Single-Copy Orthologs (BUSCO v 3.0; [36]). 96.8% of the *Ixodes* BUSCOs were identified as complete in the *T*. *perfuga* assembly. All raw sequencing reads are available in the NCBI Short Read Archive, accession number: SRP148479. The transcriptome is deposited at the DDBJ/EMBL/GenBank Transcriptome Shotgun Assembly database (accession number GGOF00000000).

### Annotation

BLASTX searches (e-value < 1e-5) to both NCBI nr and UniProtKB were used to automatically annotate transcripts [37]. Putative chimeric and contaminant sequences were removed from the resulting assemblies following Clarke et al. [38]. Functional annotation was done with Gene Ontology (GO) terms associated with the best UniProt matches. Translation of assembled contigs based on the frame of the best BLASTX hit to nr by e-value was used to generate predicted proteins. If a transcript had no BLASTX hit, amino acid sequence was predicted using the longest open reading frame (ORF) following Clarke et al. [39].

Spidroin gene family members identified from the automatic annotation were further examined with additional BLASTX searches (e-value < 1 e-5) against a protein database with spidroin genes downloaded from NCBI nr proteins and UniProtKB/Swiss-Prot databases (September 2016) in Geneious v8.1.8 [40]. Visual inspection confirmed the presence of known characteristics of spidroin genes, such as repetitive regions and coding regions for conserved N- and C- terminal domains (S2 Table). To be conservative in reporting the number of new spidroins, transcripts with pairwise nucleotide identities >95% were considered to represent the same variant and only the longest transcript was used for subsequent analyses.

### Phylogenetic and expression analyses of spidroin family members

Phylogenetic analyses of spidroin family members were done by aligning the N- and C-terminal region translations of *T*. *perfuga* spidroin contigs with published spidroin sequences from araneomorph (true spider) spiders (S3 Table). A spidroin terminal region from a non-araneomorph spider, *Bothriocyrtum californicum* (Mygalomorphae: Ctenizidae), was used to root each analysis (GenBank accessions EU117162 and HM752562). Amino acid alignments were done with MUSCLE [41] as implemented in Geneious and refined by eye. Amino acid model test and maximum likelihood gene tree construction with 10,000 bootstrap replicates were done in RAxML v8.2.8 [42]. JTT and LG likelihood amino acid substitution models were used for N- and C- terminal region alignments, respectively. Resulting trees were visualized with FigTree v1.4.3 (http://tree.bio.ed.ac.uk/software/Fig.tree/).

The relative levels of spidroin gene expression in *T*. *perfuga* silk glands were quantified by mapping filtered sequencing reads from *T*. *perfuga* RNA-Seq libraries (combination of all silk glands within individual mature females, i.e. two biological replicates) to our female *T*. *perfuga* transcriptome using TopHat2 v2.1.1 with default parameters [43]. Reads Per Kilobase per Million mapped read (RPKM) values were calculated for each spidroin transcript. Spidroins with at least ten mapped reads and one RPKM were kept for further analysis.

## Results

### *Tengella perfuga* spidroins

**Fig 1:**
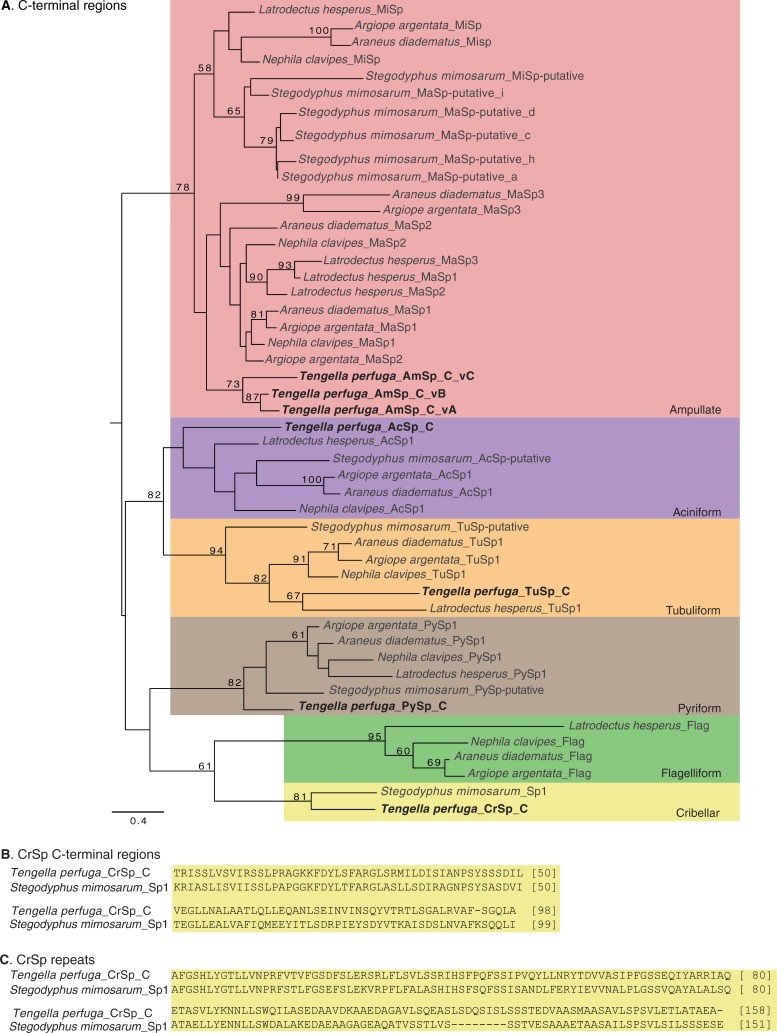
Phylogenetic analysis of *Tengella perfuga* spidroins and alignment of *T*. *perfuga* cribellar C-terminal and repeat regions with *Stegodyphus mimosarum* Spidroin 1. (**A)** C-terminal regions maximum likelihood tree. Shaded boxes indicate spidroin types, annotated as ampullate (pink), aciniform (purple), tubuliform (orange), pyriform (brown), flagelliform (green), and cribellar (yellow). Tree rooted with California trapdoor spider *Bothriocyrtum californicum* fibroin 1 (not shown). Bootstrap percentages ≥ 50% are shown. Scale bar represents substitutions per site. (**B**) C-terminal regions and (**C**) repeat regions of *T*. *perfuga* cribellar spidroin aligned with *S*. *mimosarum* Spidroin 1 (*S*.*mim*_Sp1). Gaps inserted into the alignment are indicated by dashes. Total amino acids shown on the right.

Spidroins are structural proteins composed of a large repetitive region bounded by conserved non-repetitive amino and carboxyl terminal regions [31,44]. We identified 13 spidroin contigs from *T*. *perfuga* spiders that contain N- or C- terminal coding regions and partial adjacent repetitive regions (S2 Table, S2 Fig). These spidroin contigs are associated with ampullate, aciniform, pyriform, tubuliform, and cribellate silk glands (S2 Table). Maximum likelihood analyses of the C- and N-terminal region sequences show that *T*. *perfuga* ampullate (AmSp), aciniform (AcSp), pyriform (PySp), and tubuliform (TuSp) sequences group together with spidroins of the same respective type from the comparison species (Fig 1 and S1 Fig). Within each paralog group, some spidroins grouped according to species relationships [8] such as the resolution of araneoid MiSp sequences in Fig 1. However, the relationships among spidroin homologs often did not recover species relationships, likely due to lineage specific gene duplications/loss, concerted evolution, and other sources of homoplasy.

*T*. *perfuga* has multiple ampullate spidroin variants. Six transcripts were identified as ampullate spidroins, three with the N-terminal region and the other three with the C-terminal region. Phylogenetic analyses of the terminal regions show that our ampullate sequences cluster within a diverse clade of major and minor ampullate spidroins (Fig 1 and S1 Fig). Relationships among C-terminal encoding sequences indicate that all three *T*. *perfuga* ampullate spidroin variants cluster in their own clade with moderate support (Fig 1; 73%). Similarly, all major and minor ampullate-putative termini from the cribellate spider *Stegodyphus mimosarum* also form their own clade within the ampullate group (Fig 1; 65%).

The repetitive region of *T*. *perfuga* ampullate spidroins share amino acid sequence motifs with the minor ampullate (minor ampullate spidroin-MiSp) and major ampullate (major ampullate spidroin1 and 2-MaSp1 and MaSp2) spidroins of orb-web and cob-web weaving spiders (S2 Fig). Although the repetitive regions of the *T*. *perfuga* ampullate spidroins have these motifs, the repeat sequences do not correspond to those of MiSp, MaSp1, or MaSp2. Because the repeat sequences of *T*. *perfuga* ampullate spidroins do not obviously correspond to MiSp, MaSp1, or MaSp2 of orb-web weavers, we annotated our *T*. *perfuga* ampullate-type spidroins with the general name of Ampullate Spidroin (AmSp), with a version letter to distinguish them from each other following the nomenclature used by Collin et al [45] (S2 Table).

Contrasting the multiple *T*. *perfuga* ampullate spidroins, we found evidence for only a single locus each for aciniform, pyriform, and tubuliform spidroins. *T*. *perfuga* aciniform spidroin (AcSp), the presumed main component of aciniform silk, has a similar repetitive region to the AcSp from other species [22,46–48]. *T*. *perfuga* AcSp has a repeat length of 190 amino acids (aa), which is similar to that of orb-web weavers in the genus *Argiope* (200–204 aa [22,47]) and to the two sections that compose the 375 aa aciniform spidroin repeat of the cob-web weaver *Latrodectus hesperus* (the two ~190 aa sections are alignable to each other and to the AcSp from other species, [41]). The *T*. *perfuga* AcSp repeat also has substantial serine content (24%) and the presence of poly-serine motifs (S2 Fig). Unlike previously described aciniform spidroins (e.g. [19,41–43]), the repetitive region of *T*. *perfuga* AcSp has poly-alanine amino acid motifs (S2 Fig). Indeed, poly-alanine is more prevalent and in longer stretches than poly-serine in *T*. *perfuga* AcSp.

*T*. *perfuga* PySp contains a novel combination of known PySp amino acid sequence motifs. In other species, PySp is the main component of pyriform silk, which is used to anchor silk fibers to a substrate [20,49,50]. The *T*. *perfuga* PySp sequence contains one short (10 aa) stretch of alternating prolines (PX). This amino acid motif has also been identified in PySp from the cribellate spider *S*. *mimosarum*, orb-web weaving species, and one cob-web weaving spider (*Parasteatoda tepidariorum*). In addition to PX, *T*. *perfuga* PySp also contains motifs with short runs of alanines (AAASARAEAXAR, AAASXRAA; black boxes in S2 Fig), which are similar to motifs that thus far were only found in PySp from the cob-web weaver *L*. *hesperus* (AAARAQAQAERAKAE, AAARAQAQAE; [20,51]).

*T*. *perfuga* female TuSp has extraordinary sequence conservation among the four repeat units represented in our contig, which despite being nearly 3 kb is still a partial transcript (*T*. *per_TuSp_C*). The tandem arrayed, 194 aa repeat units in *T*. *perfuga* TuSp had >94% average pairwise identity at the amino acid and nucleotide levels. This high sequence similarity among tandem repeats within a molecule has been observed in TuSp from other species [32,52]. Additionally, *T*. *perfuga* TuSp repeats are similar in length and amino acid composition, largely composed of serine and alanine, to TuSp repeats from orb-web and cob-web weaving spiders.

### Novel spidroin transcripts

*T*. *perfuga* had two novel spidroin transcripts, one containing a C-terminal region and the other an N-terminal region (*T*. *perfuga*_CrSp_C in Fig 1 and *T*. *perfuga*_Sp_N in S1 Fig). These *T*. *perfuga* transcripts had different top BLASTX hits, both of which were spidroins from the same species, the cribellate spider *S*. *mimosarum*. Because the two *S*. *mimosarum* spidroins are located on separate genome assembly scaffolds and have dissimilar repetitive region sequences, we considered our two *T*. *perfuga* spidroin transcripts as also representing separate loci.

The *T*. *perfuga* transcript containing the N-terminal region was given the name “Sp” to indicate that it is a spidroin family member, but cannot be assigned to a known category. While this transcript was placed as sister to the flagelliform clade with 50% support in the phylogenetic analysis (*T*. *perfuga*_Sp_N; S1 Fig), the repetitive sequence lacks the motifs that are characteristic of flagelliform spidroins (proline-rich motifs, intervening spacers; [53,54]).

The novel *T*. *perfuga* transcript that contained the spidroin C-terminal region was associated with cribellar silk and we thus named it *T*. *perfuga*_CrSp_C (Cribellar Spidroin). Phylogenetic analysis of C-terminal regions provides support for the annotation of this *T*. *perfuga* transcript as a CrSp. *T*. *perfuga*_CrSp_C formed a clade with *S*. *mimosarum_*Sp1 and the two C-termini shared 55% aa identity (Fig 1A and 1B). Additionally, the repetitive sequence of *T*. *perfuga*_CrSp_C and *S*. *mimosarum*_Sp1 lack motifs that are characteristic of other spidroin types, but share a novel 158 aa long repeat unit (56% identity at the aa level; Fig 1C).

### Spidroin gene expression in *T*. *perfuga* female spiders

**Fig 2:**
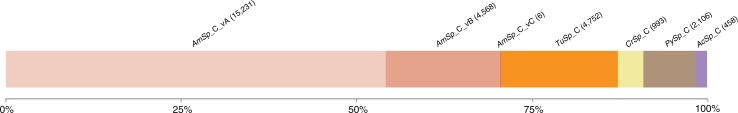
Relative silk gene expression in female *Tengella perfuga* silk glands. Silk transcripts containing the C-terminal domain are shown. Average expression from two biological replicates of *T*. *perfuga* total silk gland library reads mapped to our *de novo T*. *perfuga* transcriptome. Expression is shown as reads per kilobase of transcript per million mapped reads (RPKM, average total for each transcript shown in parentheses). Colors indicate spidroin types as in Fig 1. Names abbreviated as in S2 Table. Total RPKM of silk genes 28,114.

To investigate the relationship of silk gene expression and silk use in female *T*. *perfuga*, we compared spidroin transcript levels using RPKM of contigs containing C-terminal regions. We found spidroin transcript levels in *T*. *perfuga* spiders to be dominated (91%) by genes associated with egg sac construction (*T*. *per*_*TuSp_C*) and web construction (*T*. *per*_*CrSp_C*, *T*. *per*_*AmSp_C_vA*, *T*. *per*_*AmSp_C_vB*, and *T*. *per*_*AmSp_C_vC*). Ampullate spidroin genes (*T*. *per_AmSp_C_vA*, *T*. *per_AmSp_C_vB*, and *T*. *per_AmSp_C_vC*) were found to have the highest combined relative transcript levels compared to other spidroin genes in *T*. *perfuga* female silk glands (Fig 2). Differences in transcript levels among ampullate spidroins were also detected. Similar patterns were also observed with contigs containing N-terminal regions. One ampullate spidroin transcript (*T*. *per_AmSp_C_vA*) had the highest relative abundance when compared to other spidroins (Fig 2). We also found *T*. *per_CrSp_C*, the contig for the putative cribellar spidroin (CrSp), to account for ~ 7% of total *T*. *perfuga* silk gene expression.

## Discussion

### *Tengella perfuga* spidroins

Most of the spidroin contigs identified in this study (except for the tubuliform spidroin) contain novel combinations of amino acid sequence motifs that have not been observed before in these spidroin types (S2 Fig). The grouping of ampullate spidroins within a diverse ampullate clade has also been observed in other analyses of spidroin C-termini [44,55]. Relationships among ampullate spidroins within and across species are complicated, suggesting turnover (birth, death) and/or sequence conversion [31,32,56–58]. The repetitive regions of *T*. *perfuga* ampullate spidroins have sequence similarity to MiSp and MaSp spidroins of orb-web and cob-web weaving spiders. Similar amino acid motifs include poly-alanine (A)_n_, glycine-alanine (GA)_n_, and glycine-glycine-X (GGX)_n_, where X is a subset of all amino acids. These motifs have been related to differences in tensile properties between silk types that are primarily composed of MaSp1, MaSp2, or MiSp [59–61]. One explanation for the presence of these amino acid motifs is that *T*. *perfuga* silks have similar functional demands as the silks of orb-web and cob-web weaving spiders. In orb-web weavers, MiSp is the primary component of minor ampullate silk, which is used in the temporary spiral during orb-web construction (e.g. [62,63]). In contrast, MaSp1 and MaSp2 are the main components of major ampullate silk, which is the primary silk type in draglines and the frame and spokes of the orb-web [19,57,64]. However, *T*. *perfuga* spiders, like cob-web weavers, do not build orb-webs, and thus, the primary function of their major and minor ampullate silks is likely to be as components of the dragline.

*T*. *perfuga* aciniform and pyriform spidroins have novel combinations of motifs (S2 Fig). The presence of prevalent, long poly-alanine amino acid motifs in *T*. *perfuga* AcSp was unexpected. Poly-alanine motifs are common in other spidroins such as MaSp1 and are thought to contribute to fiber tensile strength [19,57,65–69]. It is possible that the poly-alanine in *T*. *perfuga* AcSp sequences could also increase the strength of *T*. *perfuga* aciniform silk fibers.

*T*. *perfuga* PySp is noteworthy for containing both proline (PX) and alanine-rich amino acid motifs. The PX motif found in *T*. *perfuga* PySp is shared with other spider species (e.g. *S*. *mimosarum*, *A*. *argentata*, *P*. *tepidariorum*), and has been hypothesized to provide extensibility to pyriform silk fibers in orb-web weaving spiders [70–73]. The alanine rich motifs (AAASARAEAXAR, AAASXRAA) are similar to the PySp from the cob-web weaver *L*. *hesperus* [20]. *L*. *hesperus* lacks PX, while *S*. *mimosarum*, *P*. *tepidariorum*, and orb-web weaving species lack these alanine-rich motifs [70–74]. The conservation of PX motifs and alanine-rich motifs in *T*. *perfuga* PySp sequences suggests similar functional constraints on *T*. *perfuga* PySp and PySp from orb-web and cob-web weaving spiders. Moreover, *T*. *perfuga* has the first PySp that we know of that combines the PX extensibility motif and the alanine-rich motifs in the same repeat, which has structure/function implications.

### “Modified spigot” and cribellar spidroin candidates

One of the novel spidroin transcripts, *T*. *perfuga* Sp_N, has phylogenetic affinities with flagelliform spidroins, the main component of the core fiber of the orb-web capture spiral called flagelliform silk [54]. While only ecribellate orb-web and cob-web weaving spiders have flagelliform silk glands, flagelliform spigots have been hypothesized to be homologous to pseudoflagelliform spigots, which are unique to some cribellate taxa [9,75,76]. Recently, Alfaro et al. [28] proposed that the “modified spigot” of *Tengella* is homologous to the modified/pseudoflagelliform silk spigot in other cribellate species. Thus, *T*. *per*_Sp_N may be associated with pseudoflagelliform glands. More specific annotation of this *T*. *perfuga* spidroin beyond “Sp” (e.g., as a pseudoflagelliform spidroin) requires future work to obtain more complete sequence and more closely related spidroins.

We can be more definitive about associating another novel *T*. *perfuga* spidroin with a silk gland type. Recent studies describing the web-building ontogeny of *T*. *perfuga* found that females deploy vast amounts of cribellar silk during web and retreat construction [17]. By contrast, *T*. *perfuga* males were found to use cribellar silk as juveniles and then lose the spigots associated with cribellar silks at their final molt [6,27]. Consistent with this observation, *T*. *per*_CrSp_C was present in the female tissue cDNA library constructed from the small glands attached to the spinnerets, which is where cribellar glands are expected to be located. This transcript was not present in our male (mature) tissue cDNA libraries (S2 Table). The identification of *T*. *per*_CrSp_C only in females, its distinct repeat sequence, and the placement of *T*. *per*_CrSp_C in a separate clade from the previously known spidroin types, all support that *T*. *per*_CrSp_C is a cribellar silk spidroin. In our analysis (Fig 1), CrSp orthologs are only present in the cribellate spiders *S*. *mimosarum* and *T*. *perfuga*, which suggests that CrSp has been lost in spiders that are secondarily ecribellate (without a cribellum).

### Spidroin gene expression in *T*. *perfuga* spiders

*T*. *perfuga* use multiple ampullate spidroin variants that collectively account for most of the spidroin expression in females (Fig 2). Having the highest combined relative transcript level of ampullate spidroin genes compared to other spidroin genes suggests that the ampullate spidroins are the most abundant proteins produced by *T*. *perfuga* females. The webs of *T*. *perfuga* spiders are sheet-like, with deep retreats and knockdown lines extending from overhanging substrate to the sheet below [17]. These structures are composed of at least two different silk types, with the primary silk type corresponding to dragline (ampullate) silk, and the secondary type corresponding to cribellar silk [17]. This is consistent with ampullate spidroins being the most highly expressed and cribellar spidroin expressed at a lower level (Fig 2).

The second most highly expressed spidroin type in females is TuSp, which is involved in egg case production. Female spiders wrap their egg cases mostly with tubuliform silk fibers to protect the developing embryos [32,52]. Thus, it was expected that the transcript level of *T*. *per*_*TuSp_C* would be one of the highest among spidroins in *T*. *perfuga* females (second highest, Fig 2), and absent in our male (mature) silk gland cDNA libraries given that males do not make egg cases (S2 Table).

## Conclusions

We identified 13 new spidroin contigs from the cribellate spider *T*. *perfuga*. All are partial length, seven of which are N-terminal region fragments and the other six are C-terminal region fragments (S2 Table). This means that there are at least seven spidroin genes in the *T*. *perfuga* genome. As predicted based on the presence of aciniform, ampullate, tubuliform, and pyriform silk spigots, we found *T*. *perfuga* spiders to express genes that associate with previously described aciniform, ampullate, tubuliform, and pyriform silk genes from other species. All *T*. *perfuga* spidroin types (except TuSp) have new combinations of amino acid motifs never described before for the same spidroin types from different species.

We also documented expression of a candidate cribellar spidroin, CrSp. *T*. *perfuga* is a cribellate spider, although males lose the ability to spin cribellar silk when they mature. We show evidence that *T*. *perfuga* CrSp is expressed by *T*. *perfuga* mature females but not mature males. *T*. *perfuga* CrSp has distinctive repetitive and C-terminal region sequences and gene tree analysis and pairwise alignments show an affinity with a spidroin from *S*. *mimosarum*, another cribellate species (Fig 1). Discovery of a candidate cribellate spidroin is significant as it provides insights into our understanding of the composition of cribellar silk. Furthermore, we can now begin to relate CrSp sequence to the adhesive properties of cribellar silk and trace the evolution of CrSp across different cribellate and ecribellate spider lineages.

## Supporting information

S1 Fig Maximum likelihood tree of spidroin N-terminal regions. Shaded boxes indicate spidroin types as in Fig 1. Tree rooted with California trapdoor spider *Bothriocyrtum californicum* fibroin 1 (not shown). Bootstrap percentages ≥ 50% are shown. Scale bar represents substitutions per site.
(PDF)

S2 Fig Spidroin repetitive sequences of *Tengella perfuga*. (**A**) Repetitive sequence adjacent to N-terminal region. (**B**) Repetitive sequence adjacent to C-terminal region. Spidroin names abbreviated as in S2 Table. Amino acids abundant in silks are highlighted: alanine (red), serine (blue), and glycine (green). Pyriform amino acid motifs indicated in boxes. Total number of amino acids indicated in parentheses.
(PDF)

S1 Table Summary of *Tengella perfuga de novo* transcriptome assembly. (PDF)

S2 Table *Tengella perfuga* spidroins. (PDF)

S3 Table Spidroin sequences from GenBank used in phylogenetic analyses. (PDF)

## References

[1] Bond JE, Opell BD. Testing adaptive radiation and key innovation hypotheses in spiders. Evolution 1998;52:403–14. 10.1111/j.1558-5646.1998.tb01641.x 28568335

[2] Blackledge TA, Scharff N, Coddington JA, Szüts T, Wenzel JW, Hayashi CY, et al Reconstructing web evolution and spider diversification in the molecular era. Proc Natl Acad Sci U S A 2009;106:5229–34. 10.1073/pnas.0901377106 19289848 PMC2656561

[3] Craig CL. Spiderwebs and silk: tracing evolution from molecules to genes to phenotypes Oxford University Press; 2003.

[4] Foelix R. Biology of Spiders Oxford University Press; 2011.

[5] Leister M, Mallis R, Miller K. The male of *Tengella perfuga* Dahl, 1901 with re-description of the female and comparisons with *T*. *radiata* (Kulczynski, 1909)(Araneae: Tengellidae). Zootaxa 2013;3709:185–99. 26240905 10.11646/zootaxa.3709.2.6

[6] Mallis R, Miller K. Natural history and courtship behavior in *Tengella perfuga* Dahl, 1901 (Araneae: Zoropsidae). BioOne 2017;45:166–76.

[7] Polotow D, Carmichael A, Griswold CE. Total evidence analysis of the phylogenetic relationships of Lycosoidea spiders (Araneae, Entelegynae). Invertebr Syst 2015;29:124–63. 10.1071/IS14041

[8] Fernández R, Kallal RJ, Dimitrov D, Ballesteros JA, Arnedo MA, Giribet G, et al Phylogenomics, diversification dynamics, and comparative transcriptomics across the spider tree of life. Curr Biol 2018;28:1489–97.e5. 10.1016/j.cub.2018.03.064 29706520

[9] Eberhard W, Pereira F. Ultrastructure of cribellate silk of nine species in eight families and possible taxonomic implications (Araneae: Amaurobiidae, Deinopidae, Desidae, Dictynidae, Filistatidae, Hypochilidae, Stiphidiidae, Tengellidae). J Arachnol 1993;21:161–74.

[10] Peters HM. On the spinning apparatus and the structure of the capture threads of *Deinopis subrufus* (Araneae, Deinopidae). Zoomorphology 1992;112:27–37. 10.1007/BF01632992

[11] Peters HM. Fine structure and function of capture threads In: Nentwig W. (eds) Ecophysiol. Spiders, Springer, Berlin, Heidelberg; 1987, pp. 187–202. 10.1007/978-3-642-71552-5_13

[12] Peters HM. The spinning apparatus of Uloboridae in relation to the structure and construction of capture threads (Arachnida, Araneida). Zoomorphology 1984;104:96–104.

[13] Hawthorn AC, Opell BD. van der Waals and hygroscopic forces of adhesion generated by spider capture threads. J Exp Biol 2003;206:3905–11. 10.1242/jeb.00618 14555732

[14] Hawthorn AC, Opell BD. Evolution of adhesive mechanisms in cribellar spider prey capture thread: evidence for van der Waals and hygroscopic forces: Evolution of adhesive mechanisms. Biol J Linn Soc 2002;77:1–8. 10.1046/j.1095-8312.2002.00099.x

[15] Opell BD. Factors governing the stickiness of cribellar prey capture threads in the spider family Uloboridae. J Morphol 1994;221:111–9. 10.1002/jmor.1052210109 29865401

[16] Bott RA, Baumgartner W, Bräunig P, Menzel F, Joel A-C. Adhesion enhancement of cribellate capture threads by epicuticular waxes of the insect prey sheds new light on spider web evolution. Proc R Soc B 2017;284:20170363 10.1098/rspb.2017.0363 28566485 PMC5454263

[17] Blackledge TA, Hayashi CY. Unraveling the mechanical properties of composite silk threads spun by cribellate orb-weaving spiders. J Exp Biol 2006;209:3131–40. 10.1242/jeb.02327 16888061

[18] Swanson BO, Blackledge TA, Summers AP, Hayashi CY. Spider dragline silk: correlated and mosaic evolution in high-performance biological materials. Evol Int J Org Evol 2006;60:2539–51. 17263115

[19] Hinman MB, Lewis RV. Isolation of a clone encoding a second dragline silk fibroin. *Nephila clavipes* dragline silk is a two-protein fiber. J Biol Chem 1992;267:19320–4. 1527052

[20] Blasingame E, Tuton-Blasingame T, Larkin L, Falick AM, Zhao L, Fong J, et al Pyriform spidroin 1, a novel member of the silk gene family that anchors dragline silk fibers in attachment discs of the black widow spider, *Latrodectus hesperus*. J Biol Chem 2009;284:29097–108. 10.1074/jbc.M109.021378 19666476 PMC2781455

[21] Guerette PA, Ginzinger DG, Weber BHF, Gosline JM. Silk properties determined by gland-specific expression of a spider fibroin gene family. Science 1996;272:112–5. 10.1126/science.272.5258.112 8600519

[22] Hayashi CY, Blackledge TA, Lewis RV. Molecular and mechanical characterization of aciniform silk: uniformity of iterated sequence modules in a novel member of the spider silk fibroin gene family. Mol Biol Evol 2004;21:1950–9. 10.1093/molbev/msh204 15240839

[23] Collin MA, Clarke TH, Ayoub NA, Hayashi CY. Evidence from multiple species that spider silk glue component ASG2 is a spidroin. Sci Rep 2016;6:21589 10.1038/srep21589 26875681 PMC4753498

[24] Coddington JA. Spinneret silk spigot morphology: evidence for the monophyly of orbweaving spiders, Cyrtophorinae (Araneidae), and the group Theridiidae plus Nesticidae. J Arachnol 1989;17:71–95.

[25] Gosline JM, DeMont ME, Denny MW. The structure and properties of spider silk. Endeavour 1986;10:37–43. 10.1016/0160-9327(86)90049-9

[26] Griswold CE, Ramírez MJ, Coddington JA, Platnick NI. Atlas of phylogenetic data for Entelegyne spiders (Araneae: Araneomorphae: Entelegynae), with comments on their phylogeny. Proc-Calif Acad Sci 2005;56:1.

[27] Alfaro RE, Griswold CE, Miller KB. The ontogeny of the spinning apparatus of *Tengella perfuga* Dahl (Araneae: Zoropsidae). Invertebrate Biology 2018:Forthcoming.

[28] Alfaro RE, Griswold CE, Miller KB. -Comparative spigot ontogeny across the spider tree of life. PeerJ 2018;6:e4233 10.7717/peerj.4233 29362692 PMC5772386

[29] Griswold CE, Coddington JA, Hormiga G, Scharff N. Phylogeny of the orb-web building spiders (Araneae, Orbiculariae: Deinopoidea, Araneoidea). Zool J Linn Soc 1998;123:1–99.

[30] Garb JE, DiMauro T, Lewis RV, Hayashi CY. Expansion and intragenic homogenization of spider silk genes since the Triassic: evidence from Mygalomorphae (tarantulas and their kin) spidroins. Mol Biol Evol 2007;24:2454–64. 10.1093/molbev/msm179 17728281

[31] Gatesy J, Hayashi C, Motriuk D, Woods J, Lewis R. Extreme diversity, conservation, and convergence of spider silk fibroin sequences. Science 2001;291:2603–5. 10.1126/science.1057561 11283372

[32] Garb JE, Hayashi CY. Modular evolution of egg case silk genes across orb-weaving spider superfamilies. Proc Natl Acad Sci U S A 2005;102:11379–84. 10.1073/pnas.0502473102 16061817 PMC1183556

[33] Starrett J, Garb JE, Kuelbs A, Azubuike UO, Hayashi CY. Early events in the evolution of spider silk genes. PloS One 2012;7:e38084 10.1371/journal.pone.0038084 22761664 PMC3382249

[34] Bolger AM, Lohse M, Usadel B. Trimmomatic: a flexible trimmer for Illumina sequence data. Bioinformatics 2014;30:2114–20. 10.1093/bioinformatics/btu170 24695404 PMC4103590

[35] Grabherr MG, Haas BJ, Yassour M, Levin JZ, Thompson DA, Amit I, et al Full-length transcriptome assembly from RNA-seq data without a reference genome. Nat Biotechnol 2011;29:644–52. 10.1038/nbt.1883 21572440 PMC3571712

[36] Waterhouse RM, Seppey M, Simão FA, Manni M, Ioannidis P, Klioutchnikov G, et al BUSCO Applications from Quality Assessments to Gene Prediction and Phylogenomics. Mol Biol Evol 2018;35:543–8. 10.1093/molbev/msx319 29220515 PMC5850278

[37] Altschul SF, Gish W, Miller W, Myers EW, Lipman DJ. Basic local alignment search tool. J Mol Biol 1990;215:403–10. 10.1016/S0022-2836(05)80360-2 2231712

[38] Clarke TH, Garb JE, Hayashi CY, Arensburger P, Ayoub NA. Spider transcriptomes identify ancient large-scale gene duplication event potentially important in silk gland evolution. Genome Biol Evol 2015;7:1856–70. 10.1093/gbe/evv110 26058392 PMC4524477

[39] Clarke TH, Garb JE, Hayashi CY, Haney RA, Lancaster AK, Corbett S, et al Multi-tissue transcriptomics of the black widow spider reveals expansions, co-options, and functional processes of the silk gland gene toolkit. BMC Genomics 2014;15 10.1186/1471-2164-15-365 PMC4200122 24916340

[40] Kearse M, Moir R, Wilson A, Stones-Havas S, Cheung M, Sturrock S, et al Geneious Basic: An integrated and extendable desktop software platform for the organization and analysis of sequence data. Bioinformatics 2012;28:1647–9. 10.1093/bioinformatics/bts199 22543367 PMC3371832

[41] Edgar RC. MUSCLE: multiple sequence alignment with high accuracy and high throughput. Nucleic Acids Res 2004;32:1792–7. 10.1093/nar/gkh340 15034147 PMC390337

[42] Stamatakis A. Raxml version 8: A tool for phylogenetic analysis and post-analysis of large phylogenies. Bioinformatics 2014:1312–3. 10.1093/bioinformatics/btu033 24451623 PMC3998144

[43] Kim D, Pertea G, Trapnell C, Pimentel H, Kelley R, Salzberg SL. TopHat2: accurate alignment of transcriptomes in the presence of insertions, deletions and gene fusions. Genome Biol 2013;14:R36 10.1186/gb-2013-14-4-r36 23618408 PMC4053844

[44] Garb JE, Ayoub NA, Hayashi CY. Untangling spider silk evolution with spidroin terminal domains. BMC Evol Biol 2010;10:243 10.1186/1471-2148-10-243 20696068 PMC2928236

[45] Collin MA, Clarke TH III, Ayoub NA, Hayashi CY. Genomic perspectives of spider silk genes through target capture sequencing: Conservation of stabilization mechanisms and homology-based structural models of Spidroin terminal regions. Int J Biol Macromol 2018 10.1016/j.ijbiomac.2018.02.032 29454054

[46] Ayoub NA, Garb JE, Kuelbs A, Hayashi CY. Ancient properties of spider silks revealed by the complete gene sequence of the prey-wrapping silk protein (AcSp1). Mol Biol Evol 2013;30:589–601. 10.1093/molbev/mss254 23155003 PMC3563967

[47] Chaw RC, Zhao Y, Wei J, Ayoub NA, Allen R, Atrushi K, et al Intragenic homogenization and multiple copies of prey-wrapping silk genes in *Argiope* garden spiders. BMC Evol Biol 2014;14:31 10.1186/1471-2148-14-31 24552485 PMC3933166

[48] Vasanthavada K, Hu X, Falick AM, La Mattina C, Moore AM, Jones PR, et al Aciniform spidroin, a constituent of egg case sacs and wrapping silk fibers from the black widow spider *Latrodectus hesperus*. J Biol Chem 2007;282:35088–97. 10.1074/jbc.M705791200 17921147

[49] Kovoor J, Zylberberg L. Fine structural aspects of silk secretion in a spider. II. Conduction in the pyriform glands. Tissue Cell 1982;14:519–30. 10.1016/0040-8166(82)90044-1 6890724

[50] Kovoor J, Zylberberg L. Fine structural aspects of silk secretion in a spider (*Araneus diadematus*). I. Elaboration in the pyriform glands. Tissue Cell 1980;12:547–56. 10.1016/0040-8166(80)90044-0 7434338

[51] Correa-Garhwal SM, Chaw RC, Clarke TH, Ayoub NA, Hayashi CY. Silk gene expression of theridiid spiders: implications for male-specific silk use. Zoology 2017;122:107–14. 10.1016/j.zool.2017.04.003 28536006

[52] Tian M, Lewis RV. Molecular characterization and evolutionary study of spider tubuliform (eggcase) silk protein. Biochemistry (Mosc) 2005;44:8006–12. 10.1021/bi050366u 15924419

[53] Hayashi CY, Lewis RV. Spider flagelliform silk: lessons in protein design, gene structure, and molecular evolution. Bioessays 2001;23:750–6. 10.1002/bies.1105 11494324

[54] Hayashi CY, Lewis RV. Evidence from flagelliform silk cDNA for the structural basis of elasticity and modular nature of spider silks. J Mol Biol 1998;275:773–84. 10.1006/jmbi.1997.1478 9480768

[55] Correa-Garhwal SM, Garb JE. Diverse formulas for spider dragline fibers demonstrated by molecular and mechanical characterization of spitting spider silk. Biomacromolecules 2014;15:4598–605. 10.1021/bm501409n 25340514

[56] Hayashi CY, Shipley NH, Lewis RV. Hypotheses that correlate the sequence, structure, and mechanical properties of spider silk proteins. Int J Biol Macromol 1999;24:271–5. 10342774 10.1016/s0141-8130(98)00089-0

[57] Ayoub NA, Garb JE, Tinghitella RM, Collin MA, Hayashi CY. Blueprint for a high-performance biomaterial: full-length spider dragline silk genes. PloS One 2007;2:e514 10.1371/journal.pone.0000514 17565367 PMC1885213

[58] Ayoub NA, Hayashi CY. Multiple recombining loci encode MaSp1, the primary constituent of dragline silk, in widow spiders (*Latrodectus*: Theridiidae). Mol Biol Evol 2008;25:277–86. 10.1093/molbev/msm246 18048404

[59] Sponner A, Unger E, Grosse F, Weisshart K. Differential polymerization of the two main protein components of dragline silk during fibre spinning. Nat Mater 2005;4:772–5. 10.1038/nmat1493 16184170

[60] Simmons AH, Michal CA, Jelinski LW. Molecular orientation and two-component nature of the crystalline fraction of spider dragline silk. Science 1996;271:84–7. 10.1126/science.271.5245.84 8539605

[61] Vienneau-Hathaway JM, Brassfield ER, Lane AK, Collin MA, Correa-Garhwal SM, Clarke TH, et al Duplication and concerted evolution of MiSp-encoding genes underlie the material properties of minor ampullate silks of cobweb weaving spiders. BMC Evol Biol 2017;17:78 10.1186/s12862-017-0927-x 28288560 PMC5348893

[62] Colgin MA, Lewis RV. Spider minor ampullate silk proteins contain new repetitive sequences and highly conserved non-silk-like “spacer regions”. Protein Sci Publ Protein Soc 1998;7:667–72. 10.1002/pro.5560070315 PMC2143960 9541398

[63] Chen G, Liu X, Zhang Y, Lin S, Yang Z, Johansson J, et al Full-length minor ampullate spidroin gene sequence. PLoS ONE 2012;7:e52293 10.1371/journal.pone.0052293 23251707 PMC3522626

[64] Zhang Y, Zhao A-C, Sima Y-H, Lu C, Xiang Z-H, Nakagaki M. The molecular structures of major ampullate silk proteins of the wasp spider, *Argiope bruennichi*: A second blueprint for synthesizing de novo silk. Comp Biochem Physiol B Biochem Mol Biol 2013;164:151–8. 10.1016/j.cbpb.2012.12.002 23262065

[65] Lawrence BA, Vierra CA, Moore AM. Molecular and mechanical properties of major ampullate silk of the black widow spider, *Latrodectus hesperus*. Biomacromolecules 2004;5:689–95. 10.1021/bm0342640 15132648

[66] Simmons AH, Michal CA, Jelinski LW. Molecular orientation and two-component nature of the crystalline fraction of spider dragline silk. Science 1996;271:84–7. 8539605 10.1126/science.271.5245.84

[67] Trancik JE, Czernuszka JT, Cockayne DJH, Viney C. Nanostructural physical and chemical information derived from the unit cell scattering amplitudes of a spider dragline silk. Polymer 2005;46:5225–31. 10.1016/j.polymer.2005.04.007

[68] Becker MA, Mahoney DV, Lenhert PG, Eby RK, Kaplan D, Adams WW. X-ray Moduli of Silk Fibers from Nephila clavipes and Bombyx mori Silk Polym., vol. 544, Washington, DC: American Chemical Society; 1994, pp. 185–95. 10.1021/bk-1994-0544.ch017

[69] Jenkins JE, Sampath S, Butler E, Kim J, Henning RW, Holland GP, et al Characterizing the secondary protein structure of black widow dragline silk using solid-state NMR and X-ray diffraction. Biomacromolecules 2013;14:3472–83. 10.1021/bm400791u 24024617 PMC3914425

[70] Chaw RC, Saski CA, Hayashi CY. Complete gene sequence of spider attachment silk protein (PySp1) reveals novel linker regions and extreme repeat homogenization. Insect Biochem Mol Biol 2017;81:80–90. 10.1016/j.ibmb.2017.01.002 28057598

[71] Geurts P, Zhao L, Hsia Y, Gnesa E, Tang S, Jeffery F, et al Synthetic spider silk fibers spun from pyriform spidroin 2, a glue silk protein discovered in orb-weaving spider attachment discs. Biomacromolecules 2010;11:3495–503. 10.1021/bm101002w 21053953

[72] Perry DJ, Bittencourt D, Siltberg-Liberles J, Rech EL, Lewis RV. Piriform spider silk sequences reveal unique repetitive elements. Biomacromolecules 2010;11:3000–6. 10.1021/bm1007585 20954740 PMC3037428

[73] Sanggaard KW, Bechsgaard JS, Fang X, Duan J, Dyrlund TF, Gupta V, et al Spider genomes provide insight into composition and evolution of venom and silk. Nat Commun 2014;5:3765 10.1038/ncomms4765 24801114 PMC4273655

[74] Babb PL, Lahens NF, Correa-Garhwal SM, Nicholson DN, Kim EJ, Hogenesch JB, et al The *Nephila clavipes* genome highlights the diversity of spider silk genes and their complex expression. Nat Genet 2017;49:895–903. 10.1038/ng.3852 28459453

[75] Hajer J. Notes on the spinning of the spiders *Hyptiotes paradoxus* C.L.K., 1834, and *Uloborus wakkenaerius* Latr., 1806 (Araneae: Uloboridae). Bulletin de la Société des Science Naturelles de Neuchâtel 1991;116:99–103. 10.5169/seals-89371

[76] Coddington JA. The monophyletic origin of the orb web In: Shear W.A. Ed. Spiders Webs Behav Evol. Stanf Univ Press Stanf Calif 1986 pp. 319–63.

