# Supplementary figures and images for: Silk genes and silk gene expression in the spider Tengella perfuga (Zoropsidae), including a potential cribellar spidroin (CrSp)
Source: PLoS One. 2018 Sep 20;13(9):e0203563. doi: 10.1371/journal.pone.0203563 (PMC6147414; doi:10.1371/journal.pone.0203563)

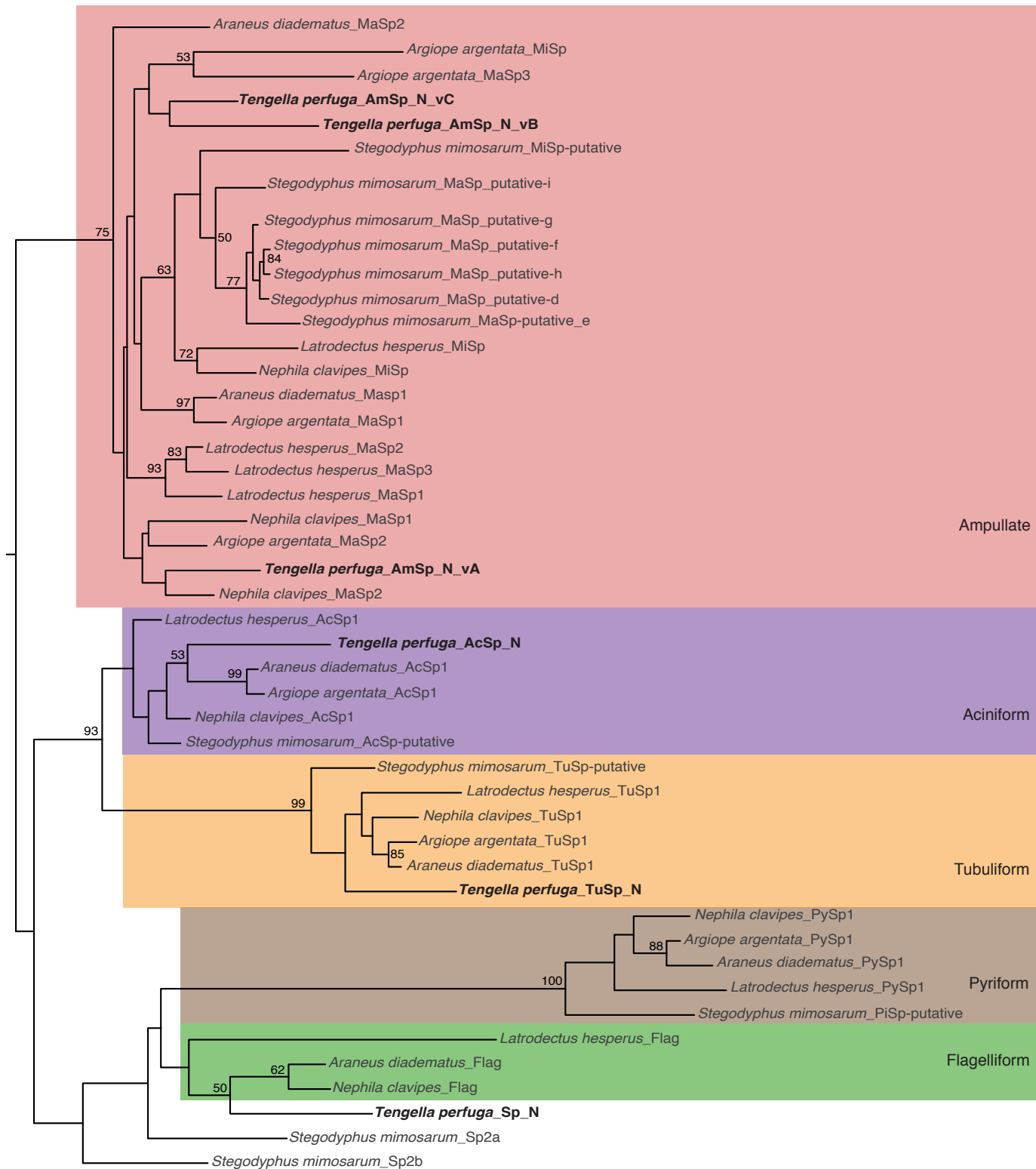

Supplement: S1 Fig — Shaded boxes indicate spidroin types as in Fig 1. Tree rooted with California trapdoor spider Bothriocyrtum californicum fibroin 1 (not shown). Bootstrap percentages ≥ 50% are shown. Scale bar represents substitutions per site. (PDF) [file pone.0203563.s001.pdf]
