# Supplementary material for: Silk genes and silk gene expression in the spider Tengella perfuga (Zoropsidae), including a potential cribellar spidroin (CrSp)
Source: PLoS One. 2018 Sep 20;13(9):e0203563. doi: 10.1371/journal.pone.0203563 (PMC6147414; doi:10.1371/journal.pone.0203563)
Supplement: S2 Fig — (A) Repetitive sequence adjacent to N-terminal region. (B) Repetitive sequence adjacent to C-terminal region. Spidroin names abbreviated as in S2 Table. Amino acids abundant in silks are highlighted: alanine (red), serine (blue), and glycine (green). Pyriform amino acid motifs indicated in boxes. Total number of amino acids indicated in parentheses. (PDF) [file pone.0203563.s002.pdf]

A. Repetitve region adjacent to N-terminal region

***T.per*_AcSp_N** (289)
YNTIDTGEQYTPPGISGSGGLYTGFGSAVKGIQAAASGAAAGSGAAGTSTTGAAAAAGASAAYGAALSSSAISSLTASVTSLLANSNDFQS
IYGGGISAQAAAQVAVGAYTSTANSLALGVSASASASVAASLRSSISNLGSGASSYAFAQAVAGSVVSGLAAAGALTSANYGSFGYVFSSA
FALASVSFASQYGIAVSSSGAASAGAAASGAAAGSGAAGTSTTTTSYSTSAASSGAAAAASGAAAAAAGASAAYGAALSSSAISSLTASVT
SLLANSNDFQSIYGGG

***T.per*_AmSp_N_vA** (66)
GPSASSSASAAGSGYGGYGSGGAGAGAGAGAGANAGAGAGAGAGRGGYGDGGAAAAAAAAAAAAGGG

***T.per*_AmSp_N_vB** (456)
TSIDTGAILNFQPKPGPSDIGQTQTGAAGGTESSAATSATNVNTTGYEPSTLLQYQTLSKVSPDGLSKYEPETRSPRTGQYTPVSGQSSQG
QTGQEIESAAVATITATSLYQVPPTPAGTAQYVPVSDPLRPGPYQTGSKNLNPVLARQGGEDIATAAATSATAAIAAVPPFTPLPPPKPGH
VQYATKPKLFRKHEYWPVSEPPSSSQSEEAGESSEAVTAAATTAEIASSSQSLPSQYEMNPTQSAVAGYPPKTEPLISGSHDPMPVHSNPP
QSQKGNEQTTVAFIDPATAAISPLPQTSSWQYQIFRQPEGPAEYALGPEPLISRQYNTWSVASSPPQSEDAEDQIAAAAATSEAVENSSPP
PFLTRQYETLSEPAGHTQNAPNPESLTTGTYDTVSVPPSRQQYEQRQVEIAAIAAASALDGISPPSPSAKRQYGALPEPPGPSQYESELEP
L

***T.per*_AmSp_N_vC**(144)
ASSRVSASSNAAVGSGVAGGVAGAGRLGGGGYGQGSGVGAGVAASGVAGAGSVGSGGYGQGAGTAAGAGQFGVSVYSQGSSAGAAAPGATA
NDVTGAGRFGGGGYGLGAGGGAGAGGSAGAGGSAGAGGGAGAGGSAGAGGGAG

***T.per*_TuSp_N** (72)
INTIPETVTRPTTAYYQAEPSTSAISTSNSFAQSSAYSLASSRAFASAFASASSASAVGSIGYNLALQTATS

***T.per*_Sp_N** (169)
SNDSDEQSITNWEKGNTQYTTRGRDEENLAVPIGADLEQRGDSGAVAGSKAQGGSKNDDDKGRFSGGSGETAEVGSPGKEGFKKGSDAQGEE
IGPEGSQQSTGRKGTANLPDELADLLDGEEGPQLSGPLLSQRGGEDGKLPDEIEAQLLGEGRSGKPTPGDGGGGSSG

B. Repetitve region adjacent to C-terminal region

***T.per*_AcSp_C** (107)
SSAFALASVSFASQYGIAVSSSGAASAGAAASGAAAGSGAAGTSTTTTSYSTSAAASGGAAAAAGASAAYGAAFGGVISSVIGGSASSGSD
LAGSSSVSIGALLSSP

***T.per*_AmSp_C_vA** (207)
GYGGYGGGDGGAAAAAAAAAAAAAAAGGAGGGYGGGGAGAGAAAAAAAAAAAAGAGGSGGAGYGGYGGYGGGDGGAAAAAAAAAAAAAAAAGG
AGGGYGGGGAGGGAGAGAAAAAAAAAAAAGAGGSGGAGYGGYGGYGGGDGGAAAAAAAAAAPAAGAGGSGGAGYGGYGGGDGGAAAAAAAAA
AAAASGGARGGRYSAPVLNTINNSV

***T.per*_AmSp_C_vB** (114)
AGAGAGAGAAGGSGGGRGGYGDGGAAAAAAAAAAAAAAAAGGAGDYGYGGYGSGGDGGAAAAAAAAAAAAAAGGARGGRGGYGGGAGASSTSS
GGRSSQRVTYSAPVLNTINNSVS

***T.per*_AmSp_C_vC** (134)
CVRSGAVAAGTAGSDIRAGRSGFQGYGQGSSAGAAAAPGAAVGSYAGAGRRGVAGFGAGAGIGVGAGSGAGAGAGAGAVAGARRRGFGVYG
QGSGAGAATAASVNAGVGRFGGGGYGQGSVGGTAVNAAVGNSV

***T.per*_PySp_C** (306)
ARAAASARAAASARAEASARAAASARAASQASSYAAARSAATTQTVTQSTTTASQSSQASAASQASAYSAARSAASSRSTAQAVSVNYQSI
QSAVSSSLSSSSALSLLSTGILSAGDIEGVVVEGLTSYGVSTANAQSVASQYLSSLGAGSSSQAYSSAIAIAVAEALSQSNVVTAGQEGYI
SEQISESISSSLSTLISQRSRPAPRPRPVPISVVSAGATPRAAASARAEALARAAASARAEASARAAASVRAAASAQAAARASSYAAASSV
GAAQTAALSSNSGRFISSGATSAASSTAVSRAS

***T.per*_TuSp_C** (860)
ASAASSAYSSSAASAQSQAAASAFSQAAAESASQAESQAASQAASQSRAFTTTSTTSEAESQASSRAASQAASRSYAAASASAFAQSSASS
LASSSAFASAFASASSASAVGSIGYNLALQTATSLGLSNAEAVASAVAQAVSNVGVGASSYAYASAVSNTVGRVLVGQGLLSQANASALAS
SFASAFASAAASASASAASSTYSSSAASAQSQAAASAFSQAAAESASQAESQAASQAASQSRAFTTTSTTSEAESQASSRAASQAASRSYA
AASASAFAQSSASSLASSSAFASAFASASSASAVGSIGYNLALQTATSLGLSNAEAVASAVAQAVSNVGVGASSYAYASAVSNTVGRVLVG
QGLLSQANASALASSFASAFASAAASASASAASSTYSSSAASAQSQAAASAFSQAAAQSASQAESQAASQAASQTRAFTSTSTTSEAESQA
SSRAASQAASHSYAAASASAFAQSSASSLASSSAFASAFASASSASAVGSIGYNLALQTATSLGLSNAEAVASAVAQAVSNVGVGASSYAY
ASAVSNTVGRVLVGQGLLSQANASTLASSFASAFASAASSASSSAASSAYSATAASAQNQAAASAFSQAASQSASQAESQSASQAASQARS
FTNTSTTSEAESQASSRAASQAASRSYAAASASAFAQSSAPSLASSSAFASAFASASSATAVGSIGYNLALQTASSLGLSNADAVASAVAQ
AVSNVGVGASSYAYASAVSKAVGRVLVGQGLLSQANASALASSFASAFASAAASASAASSAYSASGASAQSQAAASAFSQAAAQPASQATS
QFTSTGGAYDLPTIGLVAPVAPAAVAFPNFVSDMYPVLTSS

***T.per*_CrSp_C**(563)
QSEAELGVAAETRAAAVVTYVAPEDLSPSAADFARALYNRLLADAIFVRIFGTGLATEAAREYLSHMAIALSLVPAFKRVKPVKFTIAYRSS
LSSISEGADVHAYAKGIADSTATVLSRYGLVAEGGESDQASQVVTAFGSGIRKAEAAGVSTGTAATAAQETAVEQAAEIGATEEAGEEGIVG
AEVSGVSDAELQEAQAAGYAAPAGASAVESAFGRQLYAALASNRGLSLAFSRPISLLRIRGFLSGLARYIISIRQFSSLALTDLVSTYITAV
VERVTLPSTITTYLQVIVEATAEILSANRLLTREAVDAASVAVRGAASSDLVQLAEGEAGADLAAEGGAEVATATSLEGVEEAAAEGVLVTE
EAAGRGSSPAIEELGANADSLPQIASYFGLEGATDLEIAFGSHLYGTLLVNPRFVTVFGSDFSLERSRLFLSVLSSRIHSFPQFSSIPVQYL
LNRYTDVVASIPFGSSEQIYARRIAQETASVLYKNNLLSWQILASEDAAVDKAAEDAGAVLSQEASLSDQSISLSSSTEDVAASMAASAVLS
PSVLETLATAEA
