# Supplementary material for: Silk genes and silk gene expression in the spider Tengella perfuga (Zoropsidae), including a potential cribellar spidroin (CrSp)
Source: PLoS One. 2018 Sep 20;13(9):e0203563. doi: 10.1371/journal.pone.0203563 (PMC6147414; doi:10.1371/journal.pone.0203563)
Supplement: S1 Table — (PDF) [file pone.0203563.s003.pdf]

S1 Table. Summary of *Tengella perfuga de novo* transcriptome assembly

**No. Raw Paired Reads**	81,814,325
**No. Cleaned Paired Reads**	79,352,198
**No. Trinity Contigs**	127,600
**Total Length (bp)**	87,246,581
**N50 (bp)**	1,052
**BUSCO % complete**	96.8
