# Supplementary material for: Silk genes and silk gene expression in the spider Tengella perfuga (Zoropsidae), including a potential cribellar spidroin (CrSp)
Source: PLoS One. 2018 Sep 20;13(9):e0203563. doi: 10.1371/journal.pone.0203563 (PMC6147414; doi:10.1371/journal.pone.0203563)
Supplement: S2 Table — (PDF) [file pone.0203563.s004.pdf]

S2 Table. *Tengella perfuga* spidroins

Spidroin Name[a,b]	Library type	Top BLASTx Hit Accession	Top BLASTx Hit Description	E-value
*T. per*_AcSp_C	RNA-Seq of silk glands combined (Fem); cDNA of ampullate silk glands (Male)	gi|422900768|gb|AFX83561.1|	Aciniform spidroin 1, partial [*Latrodectus hesperus*]	1.10e-14
*T. per*_AcSp_N	RNA-Seq of silk glands combined (Fem)	gi|675387023|gb|KFM79920.1|	Hypothetical protein X975_02929, partial [*Stegodyphus mimosarum*]	5.15e-51
*T. per*_AmSp_N_vA	RNA-Seq of silk glands combined (Fem)	gi|295982412|pdb|3LR2|	Chain A, Self-Assembly Of Spider Silk Proteins Is Controlled By A Ph-Sensitive Relay	2.84e-25
*T. per*_AmSp_N_vB	RNA-Seq of silk glands combined (Fem)	gi|164709230|gb|ABY67420.1|	Major ampullate spidroin 1 locus 3 [*Latrodectus geometricus*]	4.07e-26
*T. per*_AmSp_N_vC	RNA-Seq of silk glands combined (Fem)	gi|295982412|pdb|3LR2|	Chain A, Self-Assembly Of Spider Silk Proteins Is Controlled By A Ph-Sensitive Relay	5.95e-41
*T. per*_AmSp_C_vA	RNA-Seq of silk glands combined (Fem); cDNA of ampullate silk glands (Fem)	gi|38197751|gb|AAR13810.1|	Major ampullate -2 [*Argiope amoena*]	4.05e-21
*T. per*_AmSp_C_vB	RNA-Seq of silk glands combined (Fem)	gi|294440291|gb|ADE74592.1|	Major ampullate spidroin 1 [*Peucetia viridans*]	1.02e-24
*T. per*_AmSp_C_vC	RNA-Seq of silk glands combined (Fem); cDNA of ampullate silk glands (Male)	gi|294440291|gb|ADE74592.1|	Major ampullate spidroin 1 [*Peucetia viridans*]	5.81e-21
*T. per*_PySp_C	RNA-Seq of silk glands combined (Fem)	gi|1148301527|gbAQR58363.1|	Pyriform spidroin 1 [*Argiope argentata*]	4.78e-11
*T. per*_CrSp_C	RNA-Seq of silk glands combined, cDNA of small silk glands attached to spinneret (Fem)	gi|675367732|gb|KFM60634.1|	Hypothetical protein X975_22661 [*Stegodyphus mimosarum*]	6.19e-71
*T. per*_Sp_N	RNA-Seq of silk glands combined (Fem)	gi|675381008|gb|KFM73910.1|	Hypothetical protein X975_01894 [*Stegodyphus mimosarum*]	1.58e-26
*T. per*_TuSp_C	RNA-Seq of silk glands combined, cDNA of tubuliform silk glands (Fem)	gi|303307781|gb|ADM14330.1|	Tubuliform spidroin 1, partial [*Agelenopsis aperta*]	4.14e-42
*T. per*_TuSp_N	RNA-Seq of silk glands combined (Fem)	gi|303307781|gb|ADM14330.1|	Tubuliform spidroin 1, partial [*Agelenopsis aperta*]	2.39e-28

[a] N or C in the spidroin names indicate whether a contig contains the N- or C- terminal region coding sequence.

[b] Variant name (e.g. _vA) does not indicate association of N- terminal transcripts with C-terminal transcripts.
