# Supplementary material for: Silk genes and silk gene expression in the spider Tengella perfuga (Zoropsidae), including a potential cribellar spidroin (CrSp)
Source: PLoS One. 2018 Sep 20;13(9):e0203563. doi: 10.1371/journal.pone.0203563 (PMC6147414; doi:10.1371/journal.pone.0203563)
Supplement: S3 Table — (PDF) [file pone.0203563.s005.pdf]

S3 Table. Spidroin sequences from GenBank used in phylogenetic analyses.

Species	Spidroin	N-terminal region Accession	C-terminal region Accession
Argiope argentata	AcSp1	AHK09813	AHK09813
Argiope argentata	Flag	--	MF955778
Argiope argentata	MaSp1	AWK58623	AWK58705
Argiope argentata	MaSp2	AWK58645	AWK58747
Argiope argentata	MaSp3	AWK58729	AWK58636
Argiope argentata	MiSp	AWK58671	AWK58662
Argiope argentata	PySp1	AQR58363	AQR58363
Argiope argentata	TuSp1	ATW75951	ATW75951
Araneus diadematus	AcSp1	AWK58687	AWK58698
Araneus diadematus	Flag	AWK58733	AWK58723
Araneus diadematus	Masp1	AWK58624	AWK58706
Araneus diadematus	MaSp2	AWK58648	AWK58752
Araneus diadematus	MaSp3	--	AWK58637
Araneus diadematus	MiSp	--	AWK58663
Araneus diadematus	PySp1	AWK58658	AWK58716
Araneus diadematus	TuSp1	AWK58641	AWK58742
Bothriocyrtum californicum	Fibroin1	HM752562	EU117162
Latrodectus hesperus	AcSp1	AFX83557	AFX83557
Latrodectus hesperus	Flag	AWK58736	AWK58725
Latrodectus hesperus	MaSp1	F595246	F595246
Latrodectus hesperus	MaSp2	F595245	F595245
Latrodectus hesperus	MaSp3	AWK58730	AWK58638
Latrodectus hesperus	MiSp	ARA91152	ARA91152
Latrodectus hesperus	PySp1	AWK58659	AWK58717
Latrodectus hesperus	TuSp1	AWK58642	AWK58744
Nephila clavipes	AcSp1	AWK58691	AWK58702
Nephila clavipes	Flag	AWK58737	AWK58726
Nephila clavipes	MaSp1	AWK58628	AWK58709
Nephila clavipes	MaSp2	AWK58653	AWK58758
Nephila clavipes	MiSp	AWK58679	AWK58667
Nephila clavipes	PySp1	AWK58660	AWK58718
Nephila clavipes	TuSp1	AWK58643	AWK58745
Nephila clavipes	TuSp1	AWK58643	AWK58745
Stegodyphus mimosarum	AcSp-putative	KFM79920	KFM79920
Stegodyphus mimosarum	MaSp-putative-a	--	KFM83271
Stegodyphus mimosarum	MaSp-putative-c	--	JT038023
Stegodyphus mimosarum	MaSp-putative-d	KFM59474	KFM59474
Stegodyphus mimosarum	MaSp-putative-e	KFM74936	--
Stegodyphus mimosarum	MaSp-putative-f	KFM61798	--
Stegodyphus mimosarum	MaSp-putative-g	KFM57717	--
Stegodyphus mimosarum	MaSp-putative-h	KFM61802	KFM61800
Stegodyphus mimosarum	MaSp-putative-i	KFM79313	KFM79313
Stegodyphus mimosarum	Misp-putative	KFM62627	KFM62627
Stegodyphus mimosarum	PiSp-putative	KFM75168	KFM68615
Stegodyphus mimosarum	Sp1	--	KFM60634
Stegodyphus mimosarum	Sp2a	KFM73910	--
Stegodyphus mimosarum	Sp2b	KFM70693	--
Stegodyphus mimosarum	TuSp-putative	KFM79920	KFM79920
